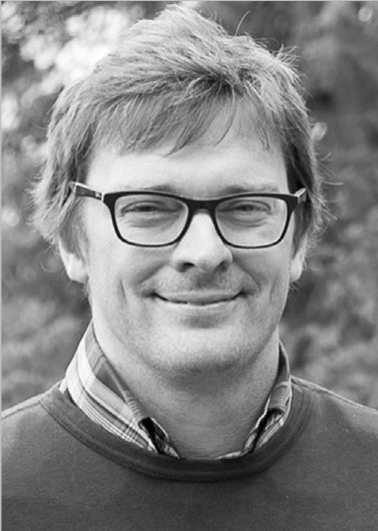# Solar Hydrogen Generation from Lignocellulose

**DOI:** 10.1002/anie.201710133

**Published:** 2018-02-05

**Authors:** Moritz F. Kuehnel, Erwin Reisner

**Affiliations:** ^1^ Christian Doppler Laboratory for Sustainable SynGas Chemistry Department of Chemistry University of Cambridge Lensfield Road Cambridge CB2 1EW UK; ^2^ Department of Chemistry Swansea University, College of Science Singleton Park Swansea SA2 8PP UK

**Keywords:** biomass, hydrogen, lignocellulose, photocatalysis, photoreforming

## Abstract

Photocatalytic reforming of lignocellulosic biomass is an emerging approach to produce renewable H_2_. This process combines photo‐oxidation of aqueous biomass with photocatalytic hydrogen evolution at ambient temperature and pressure. Biomass conversion is less energy demanding than water splitting and generates high‐purity H_2_ without O_2_ production. Direct photoreforming of raw, unprocessed biomass has the potential to provide affordable and clean energy from locally sourced materials and waste.

## 1 Introduction

Biomass is Earth's most abundant renewable resource and has been a source of energy to mankind since the Stone Age. Today, our economy depends on fossil fuels, which are derived from ancient biomass. With the gradual consumption of these non‐renewable resources and problems associated with CO_2_ emission, finding a sustainable source of energy is imperative.^1^ H_2_ is a promising energy carrier for a post‐fossil era, but current H_2_ production relies on fossil fuel reforming and is thus not sustainable.^2^ Generating H_2_ fuel directly from waste biomass without the timescales of fossilization has the potential to afford renewable energy at large scale and low cost, without competition with food production.

**Figure 1:**
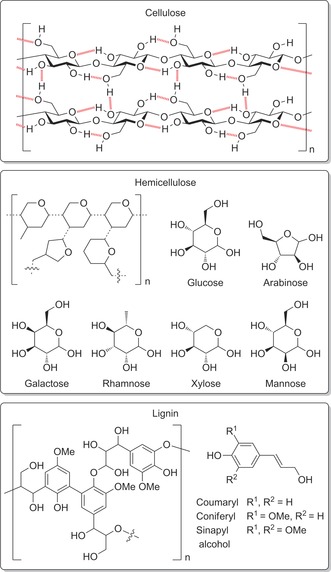
The structural components of lignocellulose.^3^

Lignocellulose is the most abundant form of biomass. It has a multi‐component structure, evolved to provide mechanical and chemical stability (Figure 1).^3^ Its primary component, cellulose, forms strong, poorly soluble fibrils comprising linear glucose β‐1,4‐homopolymer chains linked by hydrogen bonds. Cellulose fibrils are cross‐linked by hemicellulose, a branched co‐polymer of different pentose and hexose sugars. The major non‐carbohydrate component, lignin, is a polyether derived from different phenol monomers in varying compositions. It cross‐links the fibril structure and protects it from UV damage.^4^ Lignocellulose utilization is therefore kinetically challenging, as it requires disruption of this robust structure.

A number of strategies have been developed to produce fuels directly from biomass.^5^ Alcohol production from combined cellulose saccharification and fermentation is a field of intense research,^6^ but cellulose hydrolysis is slow and separation of the resulting alcohol is uneconomical at low concentrations. Thermochemical processes such as biomass gasification and reforming require high temperatures and pressures, and the generated H_2_ contains impurities that must be removed before use.^7^

## 2 Photocatalytic Reforming of Biomass

**Figure 2:**
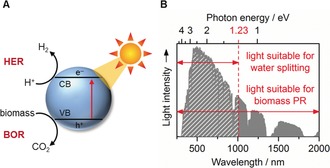
A) Photocatalytic biomass reforming process. B) The solar spectrum as it reaches the earth's surface (AM 1.5G).

Photocatalytic reforming (PR) of biomass uses the photo‐excited state of a semiconductor to drive reforming at ambient conditions (Figure 2 A). When the semiconductor absorbs light of energies greater than its band gap, an electron is excited from the valence band (VB) to the conduction band (CB). CB electrons are highly reducing and can promote the fuel‐forming hydrogen evolution reaction [HER, Eq. (1)], while the oxidizing holes left in the VB can drive the biomass oxidation reaction [BOR, shown for glucose in Eq. (2)].

H_2_ generation from water splitting [Eqs. (3) and (4)] has a large thermodynamic barrier (Δ*E* ^0^=−1.23 V) due to the energy‐demanding oxygen evolution reaction [OER, Eq. (3)]. It also generates explosive mixtures of H_2_ and O_2_. In contrast, the overall biomass reforming reaction [Eq. (5)] is almost energy neutral (Δ*E* ^0^=+0.001 V),^8^ meaning energy is only needed to overcome activation barriers. In theory, biomass PR is therefore possible using low‐energy photons (visible *and* IR light), which are highly abundant in the solar spectrum (Figure 2 B).


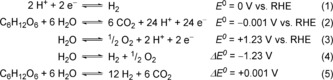


Throughout this Minireview, catalyst performance is compared on the basis of the PR rate [mmol${}_{H{}_{2}}$ g_cat_^−1^ h^−1^] and external quantum efficiency (EQE). H_2_ production is given as yield [mmol${}_{H{}_{2}}$ g_bio_^−1^].

## 3 PR of Lignocellulose Components

Photocatalytic conversion of biomass to CO_2_ and H_2_ was first reported in 1980 using TiO_2_ modified with Pt and RuO_2_ as hydrogen evolution and biomass oxidation co‐catalysts, respectively.^9^ The field has progressed significantly since then, but the majority of studies are still performed with TiO_2_‐based photocatalysts.^10^ While these materials are robust and inexpensive, their large band gaps (3.2 eV) limit solar light utilization to the UV region (Figure 2 B). PR studies initially focused on generating H_2_ from biomass‐derived feedstocks. The higher solubility and reactivity of these feedstocks facilitate reaction kinetics,^10^ but they are valuable chemicals themselves, and thus biomass PR must focus on using inedible waste material without any additional processing.

### 3.1 Sugars

Sugars have been widely studied as model substrates for biomass photoreforming, since the majority of lignocellulose is based on saccharide monomers (cellulose and hemicellulose).

Glucose PR is most established using Pt/TiO_2_.^11^ These UV light‐absorbing photocatalysts achieved performances up to 1.15 mmol${}_{H{}_{2}}$ g_cat_^−1^ h^−1^,^12^ and 8.5 % EQE.11a Other co‐catalysts (Rh,^13^ Ru,13b, 14 Pd,^15^ Au)13b, 15b, 16 showed enhanced activity, with AuPd/TiO_2_ reaching 8.8 mmol${}_{H{}_{2}}$ g_cat_^−1^ h^−1^ and 17.5 % EQE.^17^ Non‐precious co‐catalysts (Ni,15b, 18 Fe,^19^ Cu)13a gave up to 2.0 mmol${}_{H{}_{2}}$ g_cat_^−1^ h^−1^ and 59 mmol${}_{H{}_{2}}$ g_bio_^−1^ yield. Performing PR at elevated temperature (30–60 °C) improved activity15a and allowed quantitative H_2_ yield.13b, 20 Moreover, heteroatom doping (B/N,^21^ S,^22^ F)^23^ or sensitization with upconverting Er:YAlO_3_ particles was employed to improve the light absorption of TiO_2_.^24^ Pt/TiO_2_ also demonstrated PR activity towards other sugars (fructose,12c, 17, 25 galactose,^26^ mannose,26a sorbose,26a arabinose,^25^ xylose12d, 27).

Visible‐light driven glucose reforming was reported using Pt/CdZnS with rates up to 0.485 mmol${}_{H{}_{2}}$ g_cat_^−1^ h^−1^,^28^ whereas a related ZnS/ZnIn_2_S_4_ solid solution offered a lower performance.^29^ Non‐precious co‐catalysts were shown to be superior over Pt, with a MoS_2_/CdS composite^30^ achieving up to 55 mmol${}_{H{}_{2}}$ g_cat_^−1^ h^−1^ and 9.3 mmol${}_{H{}_{2}}$ g_bio_^−1^ and 81 mmol${}_{H{}_{2}}$ g_cat_^−1^ h^−1^ reported for Co/CdS/CdO_*x*_ quantum dots.^31^ Narrow‐band gap metal oxides, such as Zn:Cu_2_O (3.82 mmol${}_{H{}_{2}}$ g_cat_^−1^ h^−1^)^32^ and Fe_2_O_3_/Si (4.42 mmol${}_{H{}_{2}}$ g_cat_^−1^ h^−1^)^33^ have shown promising activities for visible‐light driven glucose PR. Other suitable materials include LaFeO_3_,^34^ Bi_*x*_Y_1‐*x*_VO_4_,^35^ CaTa_2_O_6_,^36^ La:NaTaO_3_,^37^ and SrTiO_3_.^38^

### 3.2 Oligosaccharides and Polysaccharides

Disaccharides (cellobiose,^25^, ^26^ maltose,26b, 34b sucrose,^9^, 11a, 12a,12b, 13b, 21, 26a, 39 lactose)26b generally gave lower PR rates than monosaccharides, with a maximum activity of 3.69 mmol${}_{H{}_{2}}$ g_cat_^−1^ h^−1^ reported for sucrose PR over Pt/B,N:TiO_2_ and a maximum yield of 20 mmol${}_{H{}_{2}}$ g_bio_^−1^ over Pd/TiO_2_.13b PR of soluble polysaccharides proceeded at even lower rates,^9^, 12c, 26b presumably due to their higher molecular weights and stable hydrogen‐bonded structures. Soluble starch gave 3.14 mmol${}_{H{}_{2}}$ g_cat_^−1^ h^−1^ and 26 mmol${}_{H{}_{2}}$ g_bio_^−1^ yield over Pd/TiO_2_ 13b and 1.8 % EQE over Pt/TiO_2_.11a Visible‐light driven PR of polysaccharides has only been investigated for hemicellulose with Co/CdS/CdO_*x*_, with a rate of 2.04 mmol${}_{H{}_{2}}$ g_cat_^−1^ h^−1^.^31^

### 3.3 Cellulose

Only a handful of examples have demonstrated cellulose PR. While the thermodynamics of cellulose reforming are similar to that of oligosaccharides,^40^ the kinetics are more challenging due to the compact tertiary structure of cellulose.

Direct cellulose PR was first demonstrated using Pt/TiO_2_/RuO_2_ at low activities (0.012 mmol${}_{H{}_{2}}$ g_cat_^−1^ h^−1^);^9^ comparable performance was achieved with Pt/TiO_2_.11a Improved cellulose solubility at alkaline conditions led to enhanced activity (0.041 mmol${}_{H{}_{2}}$ g_cat_^−1^ h^−1^) and 1.3 % EQE.^9^, 11b Optimization of catalyst loading, cellulose concentration, and pH further increased the performance of Pt/TiO_2_ to 0.223 mmol${}_{H{}_{2}}$ g_cat_^−1^ h^−1^.^41^ Remarkably, cellulose photoreforming proceeded with comparable activity under natural sunlight, demonstrating real‐world applicability. Immobilizing cellulose on the photocatalyst surface enhanced the rate of photocatalysis and produced 67 mmol${}_{H{}_{2}}$ g_bio_^−1^ under UV light; 14 mmol${}_{H{}_{2}}$ g_bio_^−1^ yield were produced under natural sunlight.^42^ Further enhancement was reported upon raising the reaction temperature (0.61 mmol${}_{H{}_{2}}$ g_cat_^−1^ h^−1^ at 40 °C).26b An inexpensive Ni/TiO_2_ photocatalyst achieved a performance of 0.12 mmol${}_{H{}_{2}}$ g_cat_^−1^ h^−1^ at 60 °C.15b Visible‐light driven cellulose PR was reported at Co/CdS/CdO_*x*_ in alkaline solution with rates up to 4.9 mmol${}_{H{}_{2}}$ g_cat_^−1^ h^−1^ and 7.4 mmol${}_{H{}_{2}}$ g_bio_^−1^.^31^

### 3.4 Lignin

Although lignin is considered a promising renewable feedstock,^43^ it has received little attention as a PR substrate. Lignin PR is hampered by its redox stability and brown color, limiting light absorption by the photocatalyst. Pt/TiO_2_ generated 0.026 mmol${}_{H{}_{2}}$ g_cat_^−1^ h^−1^ from lignin under UV light (0.6 % EQE).^44^ Visible‐light driven lignin PR was reported using CdS/CdO_*x*_ (0.26 mmol${}_{H{}_{2}}$ g_cat_^−1^ h^−1^)^31^ and C,N,S‐doped ZnO/ZnS.^45^

## 4 Raw Biomass PR

Direct PR of unprocessed biomass is highly desirable to lower H_2_ production cost, but is hampered by low substrate solubility. Light is scattered from insoluble biomass and absorbed by colored components. The recalcitrance of raw biomass causes a large overpotential for the BOR reaction, requiring strongly oxidizing VB holes.

PR of various plants (Table 1) was first shown over Pt/TiO_2_ at rates comparable to pure cellulose (0.004–0.018 mmol${}_{H{}_{2}}$ g_cat_^−1^ h^−1^).11a, 11b Enhanced performance was achieved in alkaline solution, or upon addition of the OER catalyst RuO_2_ (0.058 mmol${}_{H{}_{2}}$ g_cat_^−1^ h^−1^). Elevated temperatures (60 °C) allowed PR of Fescue grass over Pt/TiO_2_ at 0.061 mmol${}_{H{}_{2}}$ g_cat_^−1^ h^−1^, albeit only after removal of chlorophyll.15b Natural sunlight‐driven PR of plant matter proceeds in neutral water at rates up to 0.095 mmol${}_{H{}_{2}}$ g_cat_^−1^ h^−1^ over Pt/TiO_2_.^41^ H_2_ yields were found to vary widely across the different types of biomass (Table 1), with aquatic plants generally demonstrating higher rates and yields than terrestrial plants under similar conditions, presumably due to their lower lignin content. 3.3 mmol${}_{H{}_{2}}$ g_bio_^−1^ were produced from laver with 3.3 % EQE.11a A visible‐light absorbing Co/CdS/CdO_x_ photocatalyst showed high PR activity under simulated sunlight.^31^ Bagasse, wood, grass and sawdust gave H_2_ production rates and yields of up to 5.3 mmol${}_{H{}_{2}}$ g_cat_^−1^ h^−1^ and 0.49 mmol${}_{H{}_{2}}$ g_bio_^−1^. Strongly alkaline conditions enhanced biomass solubility and photocatalyst stability.

**Table 1:** Selected examples of photocatalytic reforming of unprocessed lignocellulose.

Substrate	Catalyst	Rate [mmol${}_{H{}_{2}}$ g_cat_^−1^ h^−1^]	Yield [mmol${}_{H{}_{2}}$ g_bio_^−1^]	EQE [%]	Conditions	Light source	Reference
cherry wood	4 % Pt/TiO_2_	0.049	0.296 (10 h)	1.1	5 m KOH	Xe	11b
wooden branch	Co/CdS/CdO_x_	5.31	0.49 (24 h)	n/a	10 m KOH, 25 °C	AM 1.5	31
sawdust	Co/CdS/CdO_x_	0.75	0.070 (24 h)	n/a	10 m KOH, 25 °C	AM 1.5	31
Dutch clover	4 % Pt/TiO_2_	0.047	0.284 (10 h)	1.1	5 m KOH	Xe	11b
goldenrod	4 % Pt/TiO_2_	0.018	0.11 (10 h)	0.4	5 m KOH	Xe	11b
rice plant	5 % Pt/TiO_2_	0.058	1.75 (10 h)	1.3	5 m KOH	Xe	11a
rice husk	0.5 % Pt/TiO_2_	0.095	n/a	n/a	H_2_O	sunlight	41
alfalfa stems	0.5 % Pt/TiO_2_	0.100	n/a	n/a	H_2_O	UV	41
turf	5 % Pt/TiO_2_	0.033	0.98 (10 h)	0.74	5 m KOH	Xe	11a
fescue grass	0.2 % Pt/TiO_2_	0.061	0.076 (3 h)	n/a	H_2_O, 60 °C	Xe	15b
grass	Co/CdS/CdO_x_	1.0	0.093 (24 h)	n/a	10 m KOH, 25 °C	AM 1.5	31
bagasse	Co/CdS/CdO_x_	0.37	0.034 (24 h)	n/a	10 m KOH, 25 °C	AM 1.5	31
water hyacinth	4 % Pt/TiO_2_	0.034	0.202 (10 h)	0.7	5 m KOH	Xe	11b
wakame seaweed	4 % Pt/TiO_2_	0.055	0.332 (10 h)	1.2	5 m KOH	Xe	11b
*chlorella* algae	5 % Pt/TiO_2_	0.090	2.7 (10 h)	2.0	5 m KOH	Xe	11a
laver	5 % Pt/TiO_2_	0.111	3.32 (10 h)	3.3	5 m KOH	Xe	11a

Biomass solubility is crucial for high PR performance. Adding detergents was shown to enhance the PR rate of castor oil at aqueous Pt/TiO_2_.^46^ PR of cotton subjected to hydrothermal liquefaction (250 °C, 40 bar)^47^ was 50 times faster than with untreated cotton under similar conditions,11b but the overall H_2_ yield was lower. Dilute acid hydrolysis of pinewood (160 °C, 10 bar) gave a hydrolysate suitable for high‐yield PR over Pt/TiO_2_ (0.813 mmol${}_{H{}_{2}}$ g_bio_^−1^).^48^ Alternatively, raw biomass can be digested at mild conditions using natural enzymes. PR of various cellulase/xylanase‐treated grasses^27^, ^49^ over Pt/TiO_2_ achieved rates up to 1.9 mmol${}_{H{}_{2}}$ g_cat_^−1^ h^−1^ and a yield of 34.6 mmol${}_{H{}_{2}}$ g_bio_^−1^. Protease A‐digested *chlorella* produced 30 mmol${}_{H{}_{2}}$ g_bio_^−1^ at rates up to 0.234 mmol${}_{H{}_{2}}$ g_cat_^−1^ h^−1[50]^ in neutral water (cf. 0.73 mmol${}_{H{}_{2}}$ g_bio_^−1^ and 0.024 mmol${}_{H{}_{2}}$ g_cat_^−1^ h^−1^ for untreated *chlorella* under these conditions).11a Although the yields and rates of pre‐treated biomass compare favorably to PR without pre‐treatment, pre‐processing adds considerable cost and time to the overall process.

## 5 The PR Mechanism

Photoreforming consists of two separate half‐reactions (see Section 2). HER is substrate‐independent, and typically proceeds at metal co‐catalysts such as Pt. This co‐catalyst acts both as a Schottky barrier that suppresses charge recombination and as a HER catalyst. PR in D_2_O has shown that the generated H_2_ originates from the aqueous solvent rather than the biomass.^11^, ^31^

**Figure 3:**
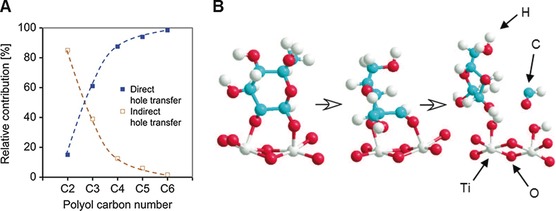
Mechanism of biomass PR on metal‐oxide surfaces. A) Mechanistic pathway depending on the substrate reproduced from Ref. 51b with permission from Elsevier. B) Mechanistic proposal for glucose reforming on TiO_2_ reproduced from Ref. 55 with permission from the ACS.

BOR is a more complex multi‐step process that directly involves the substrate. PR rates with various substrates differ depending on the substrates’ adsorption to the photocatalyst surface.11c, 12a, 13b, 28b, 42, 51 This is consistent with the Langmuir‐type kinetics observed for glucose PR on TiO_2_.13b, 15a Infrared (IR) spectroscopy,51a electron energy loss spectroscopy (EELS)51a and X‐ray absorption near edge structure (XANES)^52^ measurements confirm that glucose chemisorbs on TiO_2_. Improving this binding by changing the ionic strength,28b using α‐glucose instead of β‐glucose,^53^ or immobilizing the substrate^42^ enhances the PR rate. Chemisorption promotes electronic interactions such as substrate‐photocatalyst charge transfer,51a shifting the flat band potential11c, 12a and hole trapping at the substrate.^54^ BOR is therefore believed to involve direct hole transfer to the chemisorbed substrate (Figure 3 A),51b, 52, 54 generating surface‐bound radicals on the sub‐ns timescale, as evidenced for glucose by transient absorption spectroscopy (TAS)^52^ and electron paramagnetic resonance (EPR)^55^ spectroscopy. Fragmentation of these radicals leads to C−C bond cleavage starting from C_1_,^55^ resulting in a step‐wise degradation of glucose to arabinose, erythrose etc. with concomitant formic acid formation (Figure 3 B).13c Metal co‐catalysts can be involved in BOR, presumably by interaction with chemisorbed intermediates.51c

Alternatively, involvement of OH^.^ radicals has been suggested15b, 30, 34a, 41 on the basis of spin‐trapping EPR experiments in the absence of biomass.^14^, ^23^, ^29^ However, biomass PR is known to proceed on photocatalysts incapable of generating OH^.^ radicals.13c, 31

## 6 Biomass PR Beyond H_2_ Generation

The low market value of H_2_ renders alternative PR products desirable and, consequently, the selective photocatalytic transformation of renewable feedstocks into valuable organic products is a field of intense research.^56^ The radical nature of glucose PR over M/TiO_2_ gives rise to a number of trace by‐products such as CO,12e, 14 CH_4_,^14^, ^19^, ^22^ formic acid^16^ and others.^19^ PR of cellulose or raw biomass over Pt/TiO_2_ generated traces of C_2_H_6_, ethanol and acetone.11b Polymorph‐dependent selectivity control was observed in glucose PR over Rh/TiO_2_. Rutile showed preferred decarboxylation of glucose to give arabinose and erythrose, while further oxidation to CO_2_ was suppressed.13c LaFeO_3_ produced only H_2_ and gluconate,34b because further oxidation was slow on the less oxidizing VB compared to TiO_2_. Impregnating Pt/TiO_2_ with cellulose promoted glucose, cellobiose and formic acid formation during PR.^42^ The produced glucose could be further photoreformed at Pt/TiO_2_ to hydroxymethyl furfural.^41^ Accumulation of formate was seen during cellulose PR at CdS/CdO_*x*_,^31^ as formic acid PR was slower than cellulose PR. Formic acid could be further photoreformed at CdS to H_2_ or CO.^57^

Alternatively, reducing equivalents generated upon biomass photo‐oxidation can be used for organic transformations instead of H_2_ generation. Photocatalytic conversion of glucose to arabinose and erythrose over Pd/TiO_2_ could be coupled with the reduction of nitroarenes and aldehydes to anilines and alcohols, respectively, thus producing high‐value products from both half‐reactions.^58^ This approach was recently adapted using lignin as both reductant and oxidant.^59^ Photo‐oxidation of lignin alcohol moieties to ketones with simultaneous reductive C−O bond cleavage in the lignin backbone resulted in an overall transfer hydrogenolysis of lignin to substituted phenols.

## 7 Conclusion and Outlook

Biomass PR is a promising approach to sustainably generate fuels and feedstock chemicals. The simplicity of this room‐temperature process to produce clean H_2_ fuel is of considerable advantage over thermochemical methods, but efficiencies are yet to match conventional processes. This field has historically focused on materials and catalysts designed for solar water splitting, limiting photocatalytic activity to UV light. Future work should focus on designing narrow band‐gap materials specifically for biomass PR to enhance the performance under natural sunlight. Tailor‐made biomass oxidation catalysts will be needed to lower the required driving force and to improve the selectivity towards high‐value products. Ultimately, integrating PR with other solar fuel production systems by utilizing low‐energy photons unsuitable for water splitting may be the key to translate PR into a scalable and economically viable process.

## Conflict of interest

A patent covering biomass photoreforming has been filed by Cambridge Enterprise (PCT/EP2017/080371) that names M.F.K. and E.R. as inventors.

## Biographical Information

*Moritz F. Kuehnel received his PhD from the Freie Universität Berlin (with Dieter Lentz). He was awarded the Schering Prize for his doctoral thesis on carbon‐fluorine bond activation. After a postdoctoral stay at the HU Berlin (with Thomas Braun), he joined the group of Erwin Reisner (Cambridge) as a DFG fellow, before his promotion to Senior Postdoc. Recently, he started his independent career as a Chemistry Lecturer at Swansea University. His research encompasses the application of semiconductor nanocrystals for solar fuel production from biomass and CO_2_*.

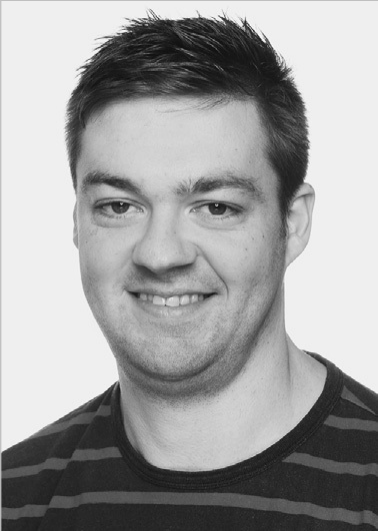

## Biographical Information

*Erwin Reisner obtained his PhD at the University of Vienna (with Bernhard K. Keppler), followed by postdoctoral research at the Massachusetts Institute of Technology (with Stephen J. Lippard) and the University of Oxford (with Fraser A. Armstrong). He is currently the Professor of Energy and Sustainability in the Department of Chemistry at the University of Cambridge, head of the Christian Doppler Laboratory for Sustainable SynGas Chemistry, and director of the UK Solar Fuels Network. His group develops solar‐driven chemistry by combining chemical biology, synthetic chemistry and materials chemistry*.